# In Memory of Sir Peter J. Lachmann, 1931–2020—Bees, Tickover, Factor I and Complement

**DOI:** 10.3390/v13081629

**Published:** 2021-08-17

**Authors:** Seppo Meri

**Affiliations:** Department of Bacteriology & Immunology, Haartman Institute (Haartmaninkatu 3), University of Helsinki, P.O. Box 21, FIN-00014 Helsinki, Finland; seppo.meri@helsinki.fi

We were all very sorry to hear that our highly esteemed colleague and one of the true pioneers of complement research, Professor, Sir Peter J. Lachmann passed away on St. Stephen’s Day, 26 December 2020 at the age of 89 years. He had worked in Cambridge, UK, as the Director of the Medical Research Council (MRC) Group on Mechanisms in Tumour Immunity.

Sir Peter was known as an intelligent person, who could challenge anyone on the history or deep knowledge about the complement system. People in complement meetings often heard accurate accounts of experiments performed in the past Figure 1. Sometimes we might have felt embarrassed to hear that our “novel” observations or thoughts were only repetitions from times 50–60 years ago or even longer. For the whole complement field it is miserable to lose the only person, who had a good grasp and personal experience of the whole history of the complement research. Nobody remembered as Peter.

Roughly 30 years ago, in 1989–90, I had the privilege of being an EMBO fellow and a post doc in Peter’s lab in Cambridge, England. In those days, Peter was the President of the Royal College of Pathologists and spent plenty of his time in London. Usually I only had 5–10 min a week to chat with him. On some lucky occasions we had longer discussions about life in general, and going through some historical events, like the work on conglutinins and immunoconglutinins by the Finnish scientist Werner Oswald Streng with Jules Bordet, 1909. Peter started his research working on lupus, but did also early complement research on a protein called conglutinogen activating factor (KAF), now known as complement factor I. Conglutinin itself, however, has almost been forgotten, at least as a human protein. It is a lectin, carbohydrate binding protein, found in bovine plasma. Oswald Streng was particularly interested in lectins. I came across this, when we found large collections of plant seeds in the basement of our Institute and wondered what they were and why were they there. It turned out that he had collected the seeds from all around the world to look for lectins that could be used e.g., for identification and classification of blood groups. Immunoconglutinins, however, probably have found themselves a new identity as C3 nephritic factors or as other autoantibodies against complement factors.

Although practicalities of actual science and experiments were discussed more with colleagues in the lab, discussion of science with Peter was very exciting. It usually occurred at a high level. Peter had strong opinions on many scientific concepts that extended beyond the field of complement. Usually we agreed, but for a young scientist it was interesting to observe that one could also have opinions that differed from a a great master. Naturally, that made one wonder who at the end was right. He was, in most cases.

One of the concepts that Peter was famous for was his tickover-hypothesis of the alternative pathway of complement. He suggested and as has more or less been nowadays been proven that the alternative pathway is constantly active at a low rate ticking over and when needed, it would get activated at higher speed. He likened it to a car engine that is on but idle, but after pressing the accelerator pedal it would really start running. This would depend on small amounts of preactive C3 (or C3b) being present and formed all the time, and factors H and I controlling the activation. Factor I (KAF) was Peter Lachmann’s special favorite. Once it was removed, the engine of the alternative pathway would turn itself on the accelerator mode. Here we disagreed a bit, because I thought that it was the role of factor H to act as the key throttle for the engine. Except of course, factors H and I work together.

In Peter’s lab, and together with Paul Morgan in Cardiff, we conducted a nice study on the mechanism of action of CD59 or protectin, as we called it in inhibiting C9 insertion into MAC. As a young scientist I wanted to submit our paper to a high impact journal, like *J. Exp. Med*. Peter, however, strongly opposed and said that it should be sent to Immunology, a journal of the British Society for Immunology, and that is what happened. Peter explained that as long as the journal is a decent one the results and story should speak for themselves, and people will find them. As a side product, we found that CD59/protectin did not inhibit killing by perforin (from NK cells and T cells), a negative result. With that, Peter said that we can do whatever we want. Thus, it was sent to *J. Exp. Med*., where it was published. Thus, at the end, we all got what we wanted.

Peter was a warm-hearted sympathetic personality, living wisely and modestly. Occasionally, however, it seemed to me as if the crowds of wild students in Cambridge had had an influence on Peter. He was joyful, curious and often with a twinkle in his eye, just like the many students enjoying their lives in Cambridge away from home. On the other the hand, he seemingly enjoyed the aristocratic traditions of the United Kingdom, life at the Christ’s College, where he occasionally invited his guests for a High Table Dinner and a glass of sherry afterwards. In his way, he was a philosopher and historian, one of the great personalities, well known and highly appreciated not only in the complement field but outside of it as well. During his career, he held many positions of trust. One of the most notable ones, close to his heart and linked with science was “Keeper of the Bees”, a special position of the Christ’s College. He worked on allergy, bee allergy especially.

Everybody remembers Peter’s and his wife’s, Sylvia’s, hospitality, when they invited students and colleagues to their home. Sylvia, another most sympathetic personality, has many characteristics similar to Peter. The two appeared inseparable and a perfect match. Our hearts feel for Sylvia, the three children (Robin, Helen and Michael) and the rest of the family. For Peter, we will never forget you and your wisdom. Thanks for explaining us our places in the historical continuum of complement research.

Seppo Meri

A Post Doc in Cambridge with Peter Lachmann (1989–1990).

## Figures and Tables

**Figure 1:**
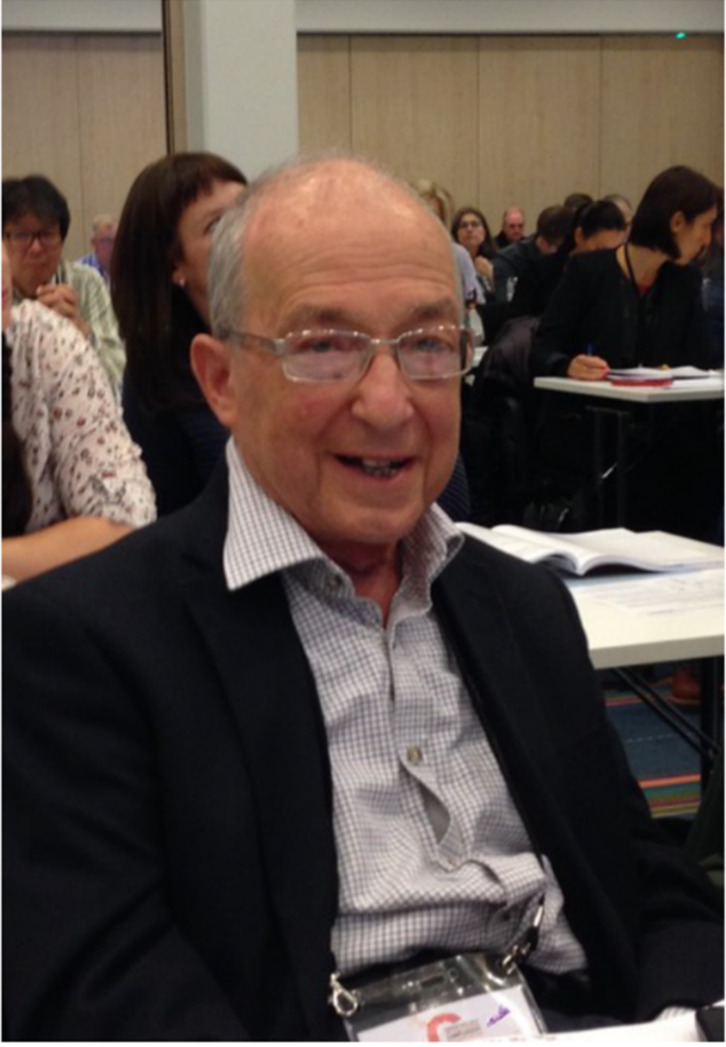
Sir Peter J. Lachmann at the European Complement Network (ECN) meeting, in Copenhagen, Denmark, 11 September 2017. Peter Lachmann was the recipient of ECN Gold Medal. Photo by Seppo Meri.

